# Incidence and Treatment of Developmental Hip Dysplasia in Mongolia: A Prospective Cohort Study 

**DOI:** 10.1371/journal.pone.0079427

**Published:** 2013-10-24

**Authors:** Bayalag Munkhuu, Stefan Essig, Erdenesuvd Renchinnyam, Raoul Schmid, Corina Wilhelm, Julia Bohlius, Battulga Chuluunbaatar, Enkhtur Shonkhuuz, Thomas Baumann

**Affiliations:** 1 National Center for Maternal and Child Health, Ulaanbaatar, Mongolia; 2 Institute of Social and Preventive Medicine (ISPM), University of Bern, Bern, Switzerland; 3 Baarer Kinderarztpraxis Baar, Baar, Switzerland; 4 Praxis, Kunterbunt, Baar, Switzerland; 5 Zentrum für körper- und sinnesbehinderte Kinder, Solothurn, Switzerland; Delft University of Technology (TUDelft), Netherlands

## Abstract

**Background:**

In Mongolia, adequate early diagnosis and treatment of developmental hip dysplasia (DDH) have been unavailable and its incidence was unknown. We determined the incidence of ultrasonographic DDH in newborns and established adequate procedures for diagnosis and treatment of DDH at the largest maternity hospital in Ulaanbaatar, Mongolia.

**Methodology/Principal Findings:**

During one year (Sept 2010 – Aug 2011) we assessed the hips newborns using ultrasound and Graf’s classification of DDH. 8,356 newborns were screened; median age at screening was 1 day. We identified 14,873 Type 1 (89.0%), 1715 Type 2a (10.3%), 36 Type 2c (0.2%), 70 Type D (0.4%), 14 Type 3 (0.08%), and 4 Type 4 hips (0.02%). Children with Type 1 hips (normal) were discharged. Children with Type 2a hips (physiologically immature) received follow-up ultrasounds at monthly intervals. Children with Type 2c to 4 (DDH; deformed or misaligned hip joint) hips were treated with a Tubingen hip flexion splint and also followed up. The hip abnormalities resolved to mature hips in all children who were followed up. There was no evidence for severe treatment related complications.

**Conclusion/Significance:**

This study suggests that the incidence of DDH in Mongolian neonates is comparable to that in neonates in Europe. Early ultrasound-based assessment and splinting treatment of DDH led to mature hips in all children followed up. Procedures are feasible and will be continued.

## Introduction

In Mongolia, adequate early diagnosis and treatment of developmental hip dysplasia (DDH), a frequent developmental deformation or misalignment of the hip joint [1], is currently not available. DDH can be cured with simple measures if adequate methods for diagnosis and treatment are available. However, in Mongolia diagnosis of DDH is currently based on clinical signs and symptoms followed up with x-ray of the hips. X-ray equipment is often outdated leading to high exposure to radiation. Diagnosis is currently usually made late, i.e. at 6 months of age. Treatment in Mongolia is based on overhead extensions for repositioning, and Pavlik harnesses with a forced abduction of up to 90 degrees, which can cause avascular necrosis of the femoral head. Thus, alternative methods for DDH screening need to be tested to reduce avoidably morbidity from DDH.

In Europe, incidence rates of hip abnormalities in unselected newborn populations indicate physiologically immature hips in 3% to 13% and deformed or misaligned hips in 1-3% of children [2-4]. The incidence of DDH in Mongolia has not been established yet.

We aimed to 1) determine the incidence of ultrasonographic detected hip abnormalities in an unselected newborn population and 2) to test the feasibility of ultrasonographic screening for DDH at the largest maternity hospital in Ulaanbaatar/Mongolia.

## Methods

### Ethics statement

We received ethical approval from the Mongolian ethical review committee, commissioned by the Ministry of Health. All parents signed a consent form. All data were anonymized before analysis.

### Sample/Procedure

In a prospective birth cohort study we evaluated the hips of all newborns at National Center for Maternal and Child Health (NCMCH), the largest maternity hospital in Mongolia, with ultrasound between September 1, 2010 and August 31, 2011. We did the baseline assessment within two days after birth, before families left the hospital. We classified hip morphology and stability according to Graf: Type 1 hips were mature; Type 2a hips were physiologically immature; Type 2c hips were dysplastic but still centered; Type D hips were dyplastic and decentered (first stage of luxation); Type 3 were luxated; and, Type 4 hips were luxated with trapped cartilage. Types 2c to 4 were summarized as DDH [5]. Hips were not classified as Type 2b, because the children in our cohort were younger than 12 weeks and the Type 2b diagnosis is restricted to children aged older than 12 weeks.

After baseline assessment the procedure followed SVUPP recommendations. Children with Type 1 hips were discharged without any further need of treatment or follow-up care. Children with Type 2a hips at baseline were followed up by ultrasound in monthly intervals at NCMCH until they matured. Children who had 2a hips during the course of follow-up were either followed up (Type 2a+), or treated if the hip insufficiently matured (Type 2a-). Children with Type 2c to 4 hips were treated with a Tubingen hip flexion splint and also followed up until fully matured. The splint was worn 24 hours per day and parents received instructions on how to fix and remove it. The Tubingen splints were reusable, easy to use, and made of a hygienic, plastic material. Children with Type 4 hips that could not be splinted were referred to the National Center for Traumatology and Orthopedics.

The screening program was done within hospital-based a Swiss-Mongolian collaboration project. In Switzerland, the Swiss Association for Pediatric Ultrasound (SVUPP) subsidized a group of physicians responsible for creating the project framework and securing funding (Swiss-Mongolian hip dysplasia project; SMOPP). Members of SMOPP trained physicians at the NCMCH to apply standardized ultrasound methods for the screening of newborns’ hips. The screening equipment consisted of a ultrasound device operating with a 7-10 MHz linear array transducer. It included a Sonofirst holding crade and transducer fixation unit (Orthopunkt, Solothurn), to prevent tilting errors. A frame grabber (Campus medicus, Klughammer GmbH, Deggendorf) captured each sonogram from the device. The frames were saved in a secure database and transmitted to experts from SMOPP in Switzerland who checked the diagnostic accuracy and management within 48 hours. Discrepancies between Mongolian and Swiss diagnoses were resolved by discussion and consensus. 

We collected information on variables known to be associated with DDH in other populations [6]: female gender, breech birth, multiple births, birth weight > 4,500 g, and family history of DDH (parents or siblings).

We performed descriptive analyses and Student’s t-tests with Stata 12.0 (Stata Corporation, Austin, Texas). In the main analysis, we used the child and not the hip as unit of analysis. If a child had hips with different morphologies, we evaluated the child based on the worst hip. In a sensitivity analysis, we used the hip as unit of analysis for initial Type 2a hips.

## Results

Of the 8,389 children born between September 1, 2010 and August 31, 2011, 33 were excluded because of congenital malformations, e.g., myelomeningocele and club feet. None of the parents declined participation in the study and 8,356 children were included. Characteristics of participants are summarized in Table **1**. A mean of 23 children were born at NCMCH per day. Their median age at ultrasound examination was 1 day (interquartile range: 0-2 days). Ninety-eight percent were born at term. Forty-nine percent were female, and 1-2% of children had other risk factors including breech births, multiple births, birth weight > 4,500 g, or had a parent or sibling with DDH.

**Table 1:** Characteristics of participants.

		N	%
Number of children born per day	mean +/- SD	23 +/- 7	
	median	23	
	interquartile range	18-27	
	range	1-51	
Age at ultrasound examination (days)	mean +/- SD	1.9 +/- 4.9	
	median	1	
	interquartile range	0-2	
	range	0-96	
Gestational Age	Term	8205	98.2
	Preterm	151	1.8
Risk factors	Female	4089	48.9
	Breech birth	165	2.0
	Multiple	92	1.1
	Weight at birth ≥ 4500g	136	1.6
	Parent or sibling with DDH	119	1.4

Abbreviations: SD, standard deviation; DDH, developmental dysplasia of the hip.

Incidence of hip types at baseline examination is shown in Table **2**. We found 14,873 mature hips (89.0%), 1,715 physiologically immature hips (10.3%), 36 dysplastic, centered hips (0.2%), 70 dysplastic, decentered hips (0.4%), 14 luxated hips (0.08%), and 4 luxated hips with trapped cartilage (0.02%). 7,110 children (85.1%) had two mature hips. 1,146 newborns (13.7%) had one or two physiologically immature hips. One hundred newborns (1.2%) had one or two DDH hips: 76 had one DDH hip; 24 had two DDH hips.

**Table 2:** Incidence of hip types at baseline.

**Type**	**Any hip^a^**	**Worse hip^b^**
	**% (n)**	**% (n)**
1	89.0 (14873)	85.1 (7110)
2a	10.3 (1715)	13.7 (1146)
2c	0.2 (36)	0.4 (30)
D	0.4 (70)	0.7 (56)
3	0.08 (14)	0.1 (12)
4	0.02 (4)	0.02 (2)
Total	100 (16712)	100 (8356)

^a^ Number of hips

^b^ Number of children; if a child had hips with different morphologies, the worse hip counted

Risk factors associated with DDH are shown in Table 3. Newborns who had a parent or sibling with DDH presented 12 times more often with a DDH hip compared to newborns whose parents and siblings had no DDH (Table **3**). The risk was increased 4.8-fold in those born in a breech position, compared to those who were not. The risk was also increased 4.8-fold in females and 2.5-fold in those with a birth weight above 4,500 g. There was no difference in risk when multiple births were compared with single births.

**Table 3:** Incidence and incidence risk ratio for type 2c to 4 hips according to risk factors present vs. not present at screening.

	**Any hip was type 2c to 4^a^**		**Worse hip was type 2c to 4^b^**	
	**Incidence**	**Incidence risk ratio**	**Incidence**	**Incidence risk ratio**
	**% (n)**	**(95% CI)**	**% (n)**	**(95% CI)**
Overall	0.7 (124)	-	1.2 (100)	-
If any risk factor from below present	1.3 (110)	7.3 (4.2-12.7)	2.0 (87)	%1.1 (3.5-11.1)
- parent or sibling with DDH	7.6 (18)	11.8 (7.3-19.1)	12.6 (15)	%1.1 (7.3-20.5)
- breech birth	3.3 (11)	4.8 (2.6-8.9)	4.8 (8)	%1.1 (2.1-8.7)
- female	1.2 (102)	4.8 (3.1-7.7)	2.0 (80)	%1.1 (2.6-6.8)
- birth weight ≥ 4500g	1.8 (5)	2.5 (1.0-6.2)	3.0 (4)	%1.1 (0.9-6.7)
- multiple	0.5 (1)	0.7 (0.1-5.2)	1.1 (1)	0.9 (0.1-6.4)

^a^ Number of hips

^b^ Number of children; if a child had hips with different morphologies, the worse hip counted

Abbreviations: CI, confidence interval; DDH, developmental dysplasia of the hip.

All newborns with two Type 1 hips were discharged without further need of treatment or follow-up care. The 1,137 newborns (99.2%) with one or two Type 2a hips and no worse hip were followed up with ultrasound at monthly intervals; in nine children (0.8%), the procedure that was actually carried out did not conform to SVUPP rules: One child was discharged, eight children were treated. 99 newborns (98%) with 2c-4 hips were treated with a Tubingen hip flexion splint; one child (1%) was discharged which was not conform with SVUPP rules.

**Figure 1:**
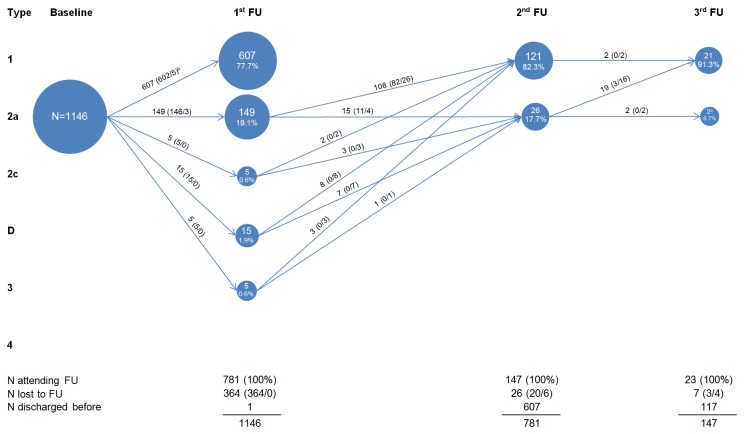
Development of type 2a hips on person level^a^. ^a^ If a child had hips with different morphologies, the worse hip counted (N=number of children); ^b^ Numbers in brackets: (X/Y) X=control, Y=treat. Abbreviation: FU, Follow-up visit; ^c^ 4^th^ FU not visualized (2 children with type 1 hips).

**Figure 2:**
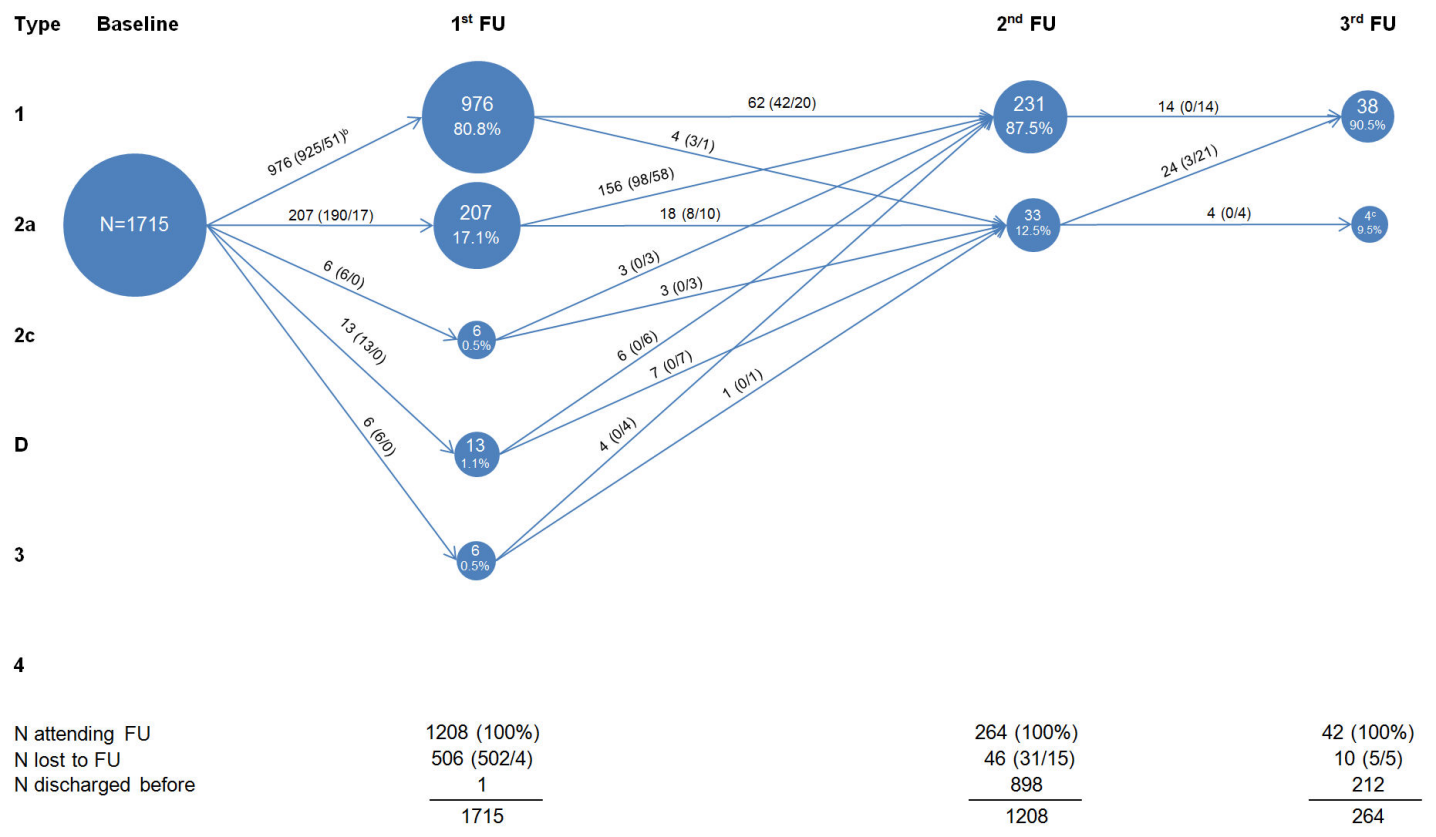
Development of type 2a hips on hip level^a^. ^a^ Any hip (N=number of hips); ^b^ Numbers in brackets: (X/Y) X=control, Y=treat. Abbreviation: FU, Follow-up visit; ^c^ 4^th^ FU not visualized (3 children with type 1 hips).

**Figure 3:**
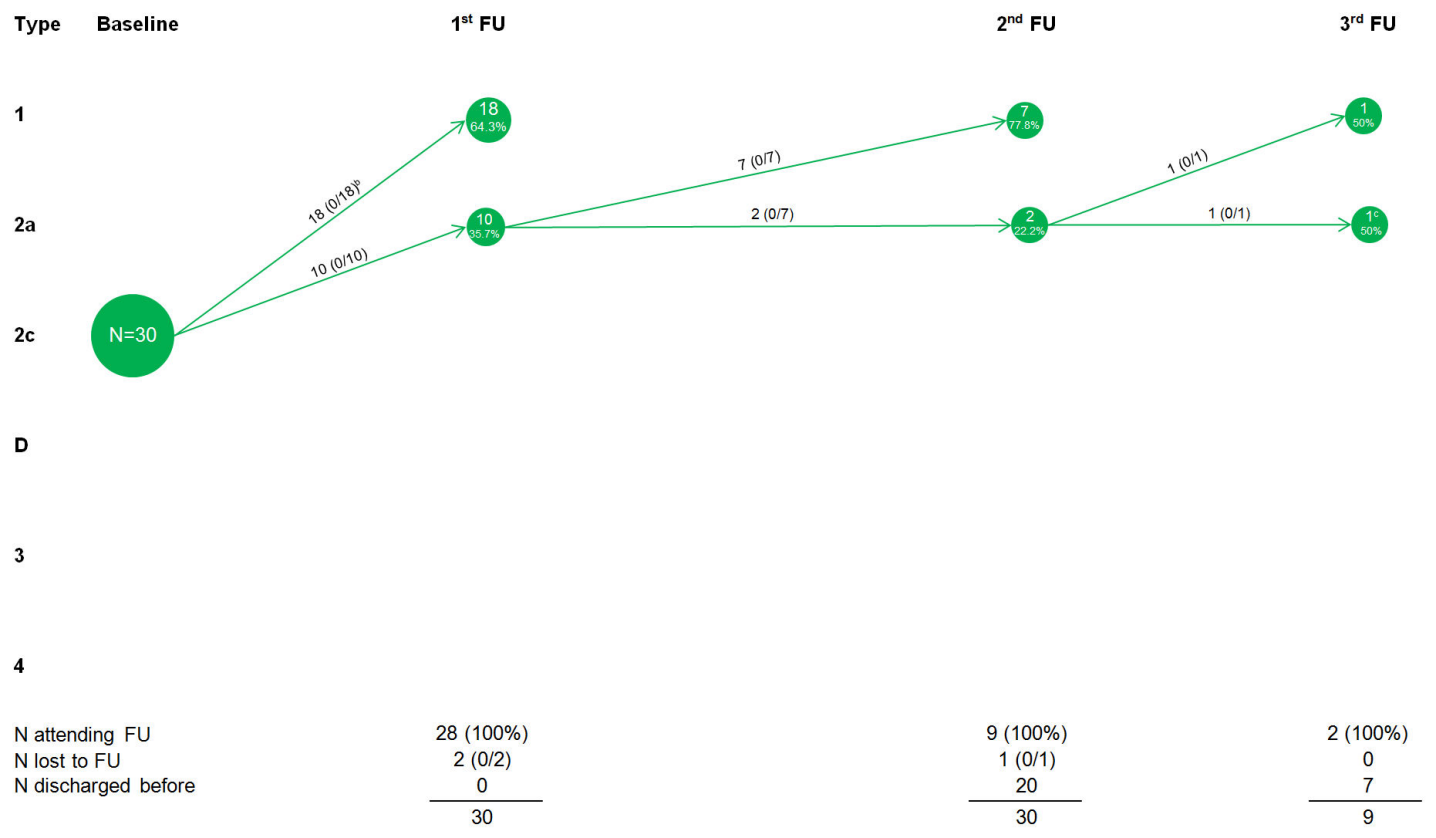
Development of type 2c hips on person level^a^. ^a^ If a child had hips with different morphologies, the worse hip counted (N=number of children); ^b^ Numbers in brackets: (X/Y) X=control, Y=treat. Abbreviation: FU, Follow-up visit; ^c^ 4^th^ FU not visualized (1 child with type 1 hips).

**Figure 4:**
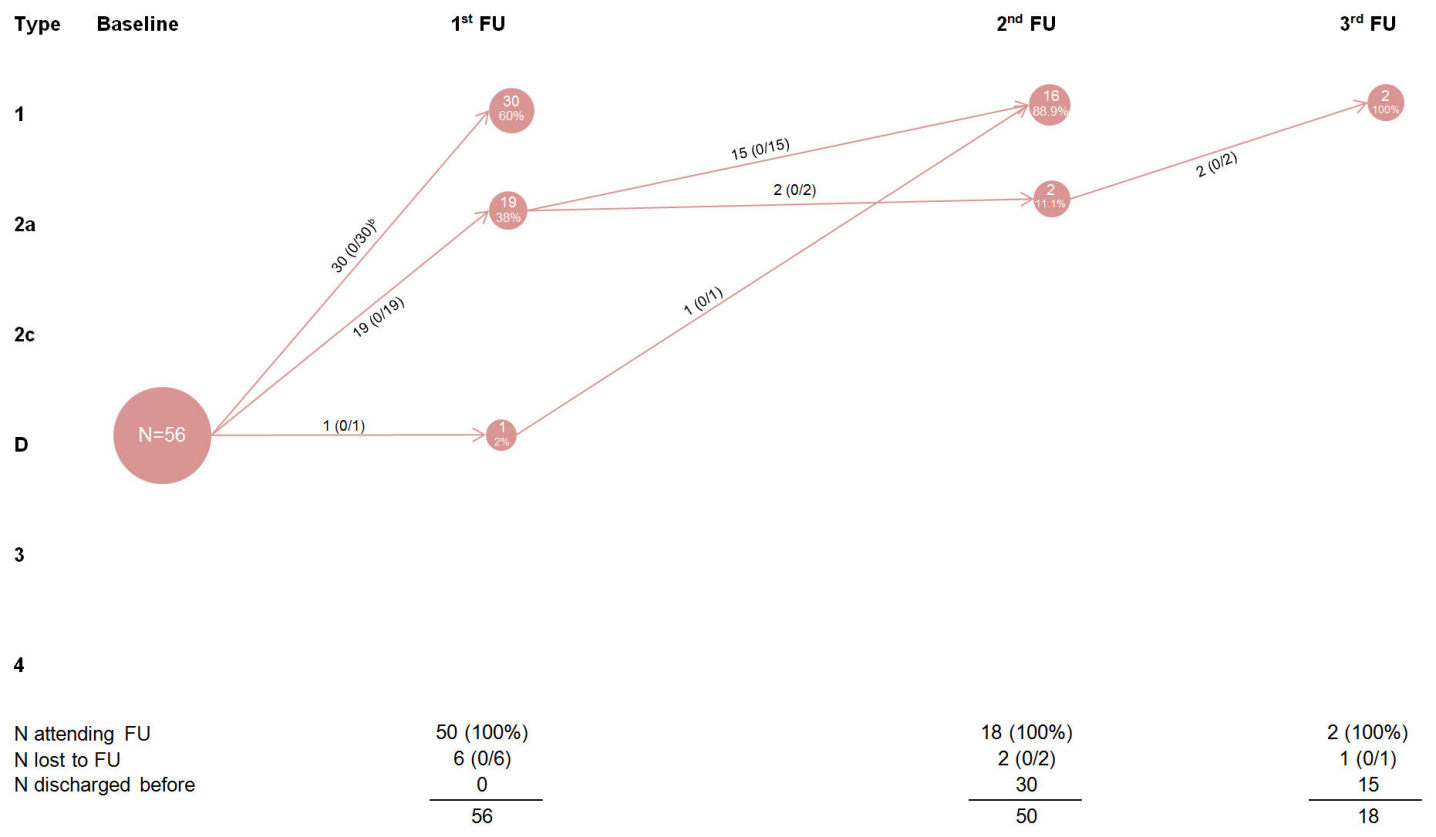
Development of type D hips on person level^a^. ^a^ If a child had hips with different morphologies, the worse hip counted (N=number of children); ^b^ Numbers in brackets: (X/Y) X=control, Y=treat. Abbreviation: FU, Follow-up visit.

Development of the hips over the course of follow-up is visualized in Figures **1**-**4** and Figures **S1**-**S2**. Of the 1146 children (68.2%) with one or two Type 2a hips and no worse hip at baseline, 781 attended a first follow-up visit (Figure **1**); 364 were lost to follow-up. Six hundred and seven of 781 (77.7%) had developed Type 1 hips; 149 (19.1%) still had Type 2a hips; and 25 (3.2%) had Type 2c-3 hips (fulfilling DDH criteria). Consequently, children with 2a hips that had insufficiently matured and all children with 2c-3 hips received follow-up treatment at first. Of the 174 children (84.5%) with type 2a-3 hips at first follow-up, 147 attended a second follow-up visit; 26 were lost to follow-up. One hundred and twenty-one of 147 (82.3%) had developed mature hips; 26 (17.7%) had physiologically immature hips. Of the 23 (91.3%) children who consecutively attended a third follow-up visit, 21 had mature hips. The two children with persistent 2a hips had mature hips within one further follow-up visit. In Figure **2**, we show development over the course of follow-up according to single 2a hips as unit of analysis at baseline. Here, those 2a hips combined with worse hips also appear. This results in an increased number of observations and a higher proportion of treated 2a hips, however, the overall result is similar to above.

Of 30 children (93.3%) with one or two type 2c hips and no worse hip at baseline, 28 attended a first follow-up visit (Figure **3**); two were lost to follow-up. Eighteen of 28 (64.3%) had developed mature hips, and 10 (35.7%) had physiologically immature hips. Nine of ten children (90%) with type 2a hips at first follow-up attended a second follow-up visit; one was lost to follow-up. Seven of nine (77.8%) had developed mature hips; 2 (22.2%) still had physiologically immature hips. One of two (50%) had matured hips at a third follow-up visit. All of these children received treatment.

Of the 56 children (89.3%) with one or two Type D hips and no worse hip at baseline, 50 attended a first follow-up visit (Figure **4**); six were lost to follow-up. Thirty of 50 (60%) had developed mature hips, 19 (38%) had physiologically immature hips, and 1 child (2%) still had a type D hip. Within a second and third follow-up visit, all children developed mature hips. All of these children received treatment.

Ten of the 12 children (83.3%) with one or two Type 3 hips and no worse hip at baseline attended a first follow-up visit (Figure **S1**); two were lost to follow-up. Two of ten (20%) had developed mature hips; eight (80%) had physiologically immature hips. Five of eight children (62.5%) with Type 2a hips at first follow-up attended a second follow-up visit; three were lost to follow-up. Four of five (80%) had developed mature hips; one (20%) still had physiologically immature hips. All of these children received treatment. We cannot report the result of the planned procedure in those two patients with one or two Type 4 hips at baseline (Figure **S2**). The family of one child declined treatment and asked for discharge, despite detailed information. In the second child, trapped cartilage made a reposition by Tubingen splint impossible. However, the local orthopedists were not able to offer a different treatment strategy before 7 months of age, so that the child was transferred to Switzerland and operated on both hips, followed by cast and later splint fixation. The treatment seems to be successful, though is not yet finished.

Overall, 188 of 8356 (2.2%) children received treatment (Table **4**). 107 (1.3%) initiated treatment after baseline assessment, and 81 (0.9%) initiated treatment during follow-up. A small proportion of children who were assigned to receive treatment had to continue treatment after follow-up visit 1 (n=40), visit 2 (n=6), and visit 3 (n=3).

**Table 4:** Decision on procedure at baseline and follow-up visits.

**Time point**	**Discharge**	**Control**	**Treatment**		**Total**
			**Newly treated**	**Already treated**	
	**% (n)**	**% (n)**	**% (n)**	**% (n)**	**% (n)**
Baseline	85.1 (7112)	13.6 (1137)	1.3 (107)	-	100 (8356)
1st Follow-up	75.8 (659)	13.0 (113)	6.6 (57)	4.6 (40)	100 (869)
2nd Follow-up	79.9 (143)	3.4 (6)	13.4 (24)	3.3 (6)	100 (179)
3rd Follow-up	85.7 (24)	3.6 (1)	0.0 (0)	10.7 (3)	100 (28)
**Treatment overall**			2.2 (188)		100 (8356)

The median time interval between baseline and the first, second and third follow-up visit was 33, 64 and 95 days, respectively (Table **5**). The mean was higher for all three intervals compared to the median time interval, documenting a skewed distribution. The mean time interval was a bit smaller in 2c-4 hips compared with 2a hips, and in bilateral compared to unilateral 2c-4 hips, without being statistically significant (37 vs. 42 days, p=0.13; 33 vs. 38 days, p=0.23; respectively). There was no evidence for severe treatment related complications.

**Table 5:** Intervals between baseline and follow-up visits.

**Intervals**		**All children (days)**	**Worse hip at screening 2c-4 (days)**	**Both hips at screening 2c-4 (days)**
**baseline - 1^st^ follow-up exam**	mean +/- SD	41 +/- 28	37 +/- 16	33 +/- 6
	median	33	33	31
	interquartile range	30-41	29-35	29-35
	range	11-290	23-110	28-53
**baseline - 2^nd^ follow-up exam**	mean +/- SD	72 +/- 33		
	median	64		
	interquartile range	58-74		
	range	32-359		
**baseline - 3^rd^ follow-up exam**	mean +/- SD	112 +/- 45		
	median	95		
	interquartile range	81-126		
	range	59-224		

Abbreviation: SD, standard deviation.

## Discussion

We found an incidence of ultrasonographically detected DDH of 0.7% in Mongolian newborns including 0.2% Type 2c, 0.4% Type D, 0.08% Type 3 and 0.02% Type 4 hips. In all children who were treated with a Tubingen hip flexion splint within the first few weeks of life, hip abnormalities resolved to Type 1. There was no evidence for severe treatment related complications.

This is the first study on the incidence of DDH in newborns in Mongolia. Our results are based on a large birth cohort comprising approximately 14% of newborns in Mongolia (8389/57020 newborns during one year) [7,8]. Mongolia has a centralized health care system and despite the size of the country; most women (44%) from across the country give birth in a hospital in Ulaanbaatar like NCMCH [9]. It is also the first screening in Asia on DDH in newborns using Graf’s method. Our study proofs the feasibility of ultrasound based screening in Mongolia. Physicians in Mongolia were trained to use and interpret sonograms following standardized procedures. With state-of-the-art software sonograms were captured and transmitted to experts located in Switzerland. Using this telemedicine, diagnoses could be validated and treatment recommendations discussed on a daily base.

The following limitations have to be addressed: Loss to follow-up in our study was high compared to European studies. However, our study included several thousand participating families in a vast country and journeys to the hospital were exhausting for parents and patients. Unfortunately, we do not know how the hips of those lost to follow-up evolved. There were some protocol violations: some examinations took place later than recommended in the SVUPP guidelines and a small proportion of procedures carried out did not conform to SVUPP recommendations.

The DDH incidence identified in our study in Mongolia is comparable to incidence rates reported in unselected newborn populations in Europe who were also examined within the first few days of delivery [2-4]. With the exception of multiple births the risk factors identified in our study were similar to the risk factors reported in the literature [2-4,6]. The quality of treatment and treatment outcomes were comparable to those reported for children in resource-rich countries [10]. A recent study in Turkey observed worsening of Type 2a hips and therefore recommends a close follow-up for patients with an initial Type 2a diagnosis [11]. This recommendation is supported by the findings from our study: We found that 25 initially controlled Type 2a hips evolved into Type 2c-3 hips at first follow-up. As in the study reported from Turkey [11] all of these hips recovered with adequate treatment until second follow-up. In our birth cohort all patients with a 2c to 4 type hip have been treated and therefore we cannot assess whether or not these hips would have resolved spontaneously. Moreover, our study does not include controls not receiving treatment to allow such an assessment. 

Whether, how and when to screen for DDH in newborns is still a matter of debate. Some authors argue that the benefit is unclear because neonatal DDH can spontaneously resolve and the effects of long term outcomes in adulthood have not been established. Others claim that only a selected sub-cohort of all newborns should be evaluated with ultrasound [10,12-15]. Clarke et al. could confirm that selective ultrasound is a successful surveillance strategy and has economic advantages compared to comprehensive screening [15]. However, the results and recommendations generated in European and North American studies cannot be transferred to Mongolia. Europe is mainly using the Graf technique, however, America the Harcke technique, which makes results difficult to compare. In Mongolia the currently provided standard care to diagnose and treat DDH is unreliable and potentially harmful, both in terms of exposure to radiation and treatment complications. Diagnosis of DDH is based on a clinical examination for asymmetric gluteal folds and the Ortolani test. However, neither are reliable tests of hip abnormalities [16,17]. Patients with positive test results receive plain film radiography at follow-up visits. Unfortunately, the x-ray equipment is often outdated and projection errors are not always accounted for. In Mongolia most children are at least 6 months old when they are diagnosed with DDH. Standard care consists of overhead extensions for repositioning, and Pavlik harnesses with a forced abduction of up to 90 degrees, which can cause avascular necrosis of the femoral head. Moreover, if a child needs hip surgery, standards are usually not sufficient to allow complex interventions in infants and toddlers in Mongolia. 

The earliest possible diagnosis of DDH in newborns allows early and effective treatment. Hips are still highly potent in ossification within the first three months of life [5]. From research in Western Europe, we know that dynamic examination after Graf for early detection which also includes a stress test in borderline cases, and treatment of hip abnormalities in newborns reduces the incidence of undetected DDH later on in life which would require open reduction [18]. In Norway, it has been estimated that 9% of all primary hip replacements are caused by DDH [19].Our study’s successful treatment and follow-up strategy is supported by a recent publication by Tschauner et al. that concluded that “ultrasound screening based-treatment of decentered hip joints has become safer, shorter and simpler: ‘‘safer’’ means lower rate of avascular necrosis, ‘‘shorter’’ means less treatment time due to earlier onset and ‘‘simpler’’ means that the devices are now less invasive and highly standardized” [20].

In terms of sustainability and capacity building our project may serve as a role model for the development of newborn screening programs in the Asia Pacific region [21]. An initial training phase was provided by pediatricians from Switzerland. Training of physicians in other hospitals in other regions of the country is now led by Mongolian pediatricians. Swiss experts will be available as advisors and continue to visit the country every year.

**Figure 5:**
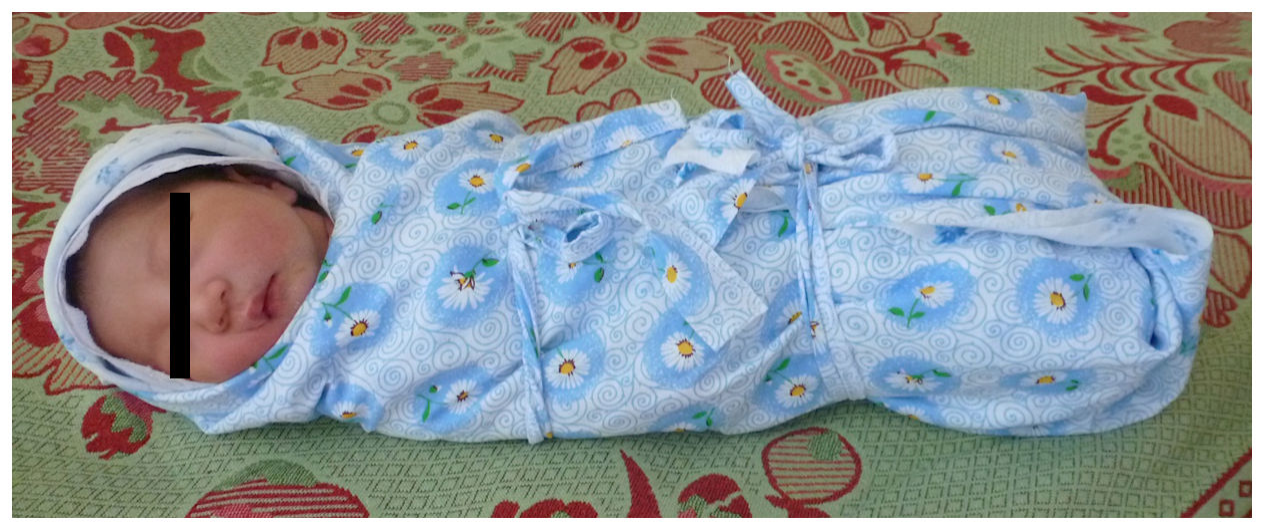
Traditional swaddling in Mongolia. The legal guardian of the subject in the photograph has given written informed consent, as outlined in the PLoS consent form, to publication of their photograph.

In a nested case-control design, we will evaluate the role of swaddling on the unsteady development of Type 2a hips which led to treatment initiation during follow-up. This is topical and important, especially in Mongolia where swaddling is common (Figure **5**). We will continue to monitor the patients enrolled in our cohort over the next years and collect information on hip development. This will lay the ground for a study on outcomes later in the lives of our participants. We will establish outpatient clinics to facilitate participation for families who live in rural provinces far from Ulaanbaatar. With the expansion of the study to other hospitals and regions of the country, we aim to demonstrate that a population-based ultrasound screening program for DDH in children is feasible and can improve the health of Mongolians.

## Conclusion

This study suggests that the incidence of DDH in Mongolian neonates is comparable to that in Europe. Early ultrasound-based assessment and splinting treatment of DDH led to mature hips in all children followed up. Procedures are feasible and will be continued.

## Supporting Information

Figure S1 **Development of type 3 hips on person level.**
(DOCX)

Figure S2 **Development of type 4 hips on person level.**
(DOCX)
