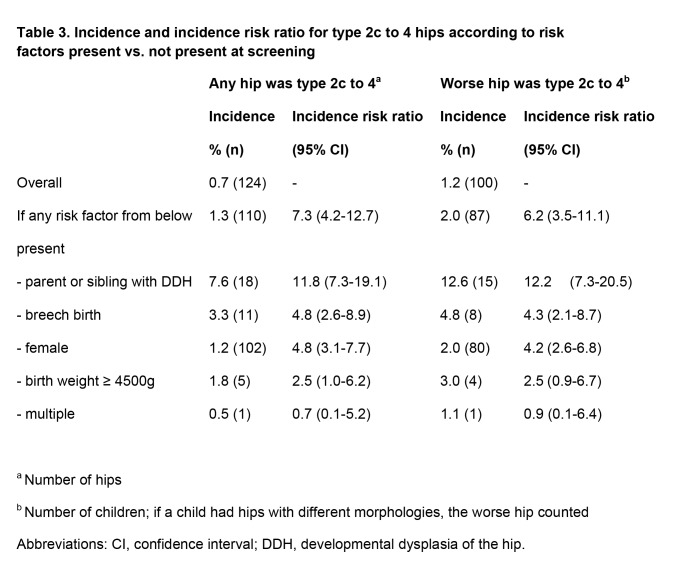# Correction: Incidence and Treatment of Developmental Hip Dysplasia in Mongolia: A Prospective Cohort Study 

**DOI:** 10.1371/annotation/3208b134-74de-4884-bd5a-0eb2e6c878ec

**Published:** 2014-01-23

**Authors:** Bayalag Munkhuu, Stefan Essig, Erdenesuvd Renchinnyam, Raoul Schmid, Corina Wilhelm, Julia Bohlius, Battulga Chuluunbaatar, Enkhtur Shonkhuuz, Thomas Baumann

An error in the production process caused the incidence risk ratios in the rightmost column of Table 3 to be displayed incorrectly. Please see the corrected Table 3 here: 

**Figure:**