# Supplementary material for: Incidence and Treatment of Developmental Hip Dysplasia in Mongolia: A Prospective Cohort Study
Source: PLoS One. 2013 Oct 24;8(10):e79427. doi: 10.1371/journal.pone.0079427 (PMC3812003; doi:10.1371/journal.pone.0079427)

**Supporting Figure S1. Development of type 3 hips on person level^a^**

^a^ If a child had hips with different morphologies, the worse hip counted (N=number of children);
^b^ Numbers in brackets: (X/Y) X=control, Y=treat.


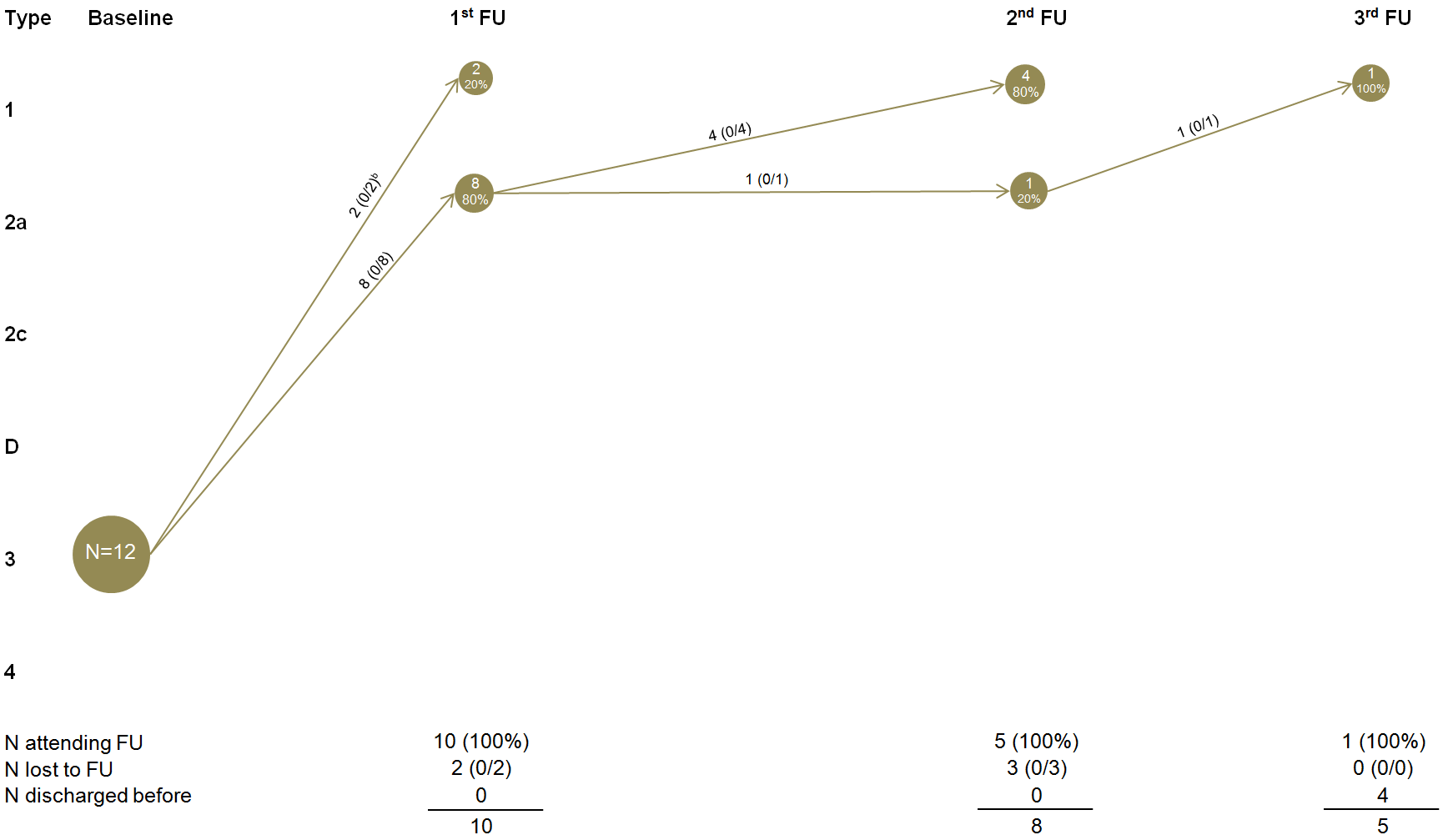

Supplement: Figure S1 — Development of type 3 hips on person level. (DOCX) [file pone.0079427.s001.docx]
